# Investigating the Experiences and Self‐Reported Abilities of Diagnostic Radiographers in Responding to Contrast‐Induced Reactions Associated With Computed Tomography Examinations: A Cross‐Sectional Survey

**DOI:** 10.1002/hsr2.71736

**Published:** 2026-01-11

**Authors:** Vivian Della Atuwo‐Ampoh, Paul Kasem, Patience Addo, Bismark Ofori‐Manteaw, Justice Hanson, David Adedia, Nathaniel Awentiirin Angaag, Jacob Leonard Ago, Klenam Dzefi‐Tettey

**Affiliations:** ^1^ Department of Medical Imaging, School of Allied Health Sciences University of Health and Allied Sciences Ho Ghana; ^2^ Faculty of Science and Health Charles Sturt University Wagga Australia; ^3^ Department of Basic Sciences, School of Basic and Biomedical Sciences University of Health and Allied Sciences Ho Ghana; ^4^ Department of Radiology Holy Family Catholic Polyclinic – Kulmasa Kulmasa Ghana; ^5^ Discipline of Medical Radiations, School of Health and Biomedical Sciences RMIT University Bundoora Campus Australia; ^6^ Radiology Department Korle‐Bu Teaching Hospital – Korle‐Bu Korle‐Bu Ghana; ^7^ Department of Radiology, School of Medicine University of Health and Allied Sciences Ho Ghana

**Keywords:** computed tomography, contrast media, diagnostic radiographers, hypersensitivity reactions

## Abstract

**Background and Aim:**

Radiographers play a crucial role in preparing and administering contrast media (CM) during computed tomography (CT) examinations, including ensuring that appropriate prophylactic pre‐medication is provided to patients at increased risk of contrast‐induced hypersensitivty reactions. Despite this responsibility, limited research has explored radiographers' perceptions and experiences in recognising and managing common hypersensitivity reactions related to CM during CT examinations. This study aims to investigate the experiences and self‐reported abilities of diagnostic radiographers in responding to contrast‐induced hypersensitivity reactions associated with CT examinations.

**Method:**

This study employed a quantitative cross‐sectional design involving licensed radiographers who perform CT examinations. The study was a survey in which a questionnaire was administered and completed by the radiographers, including demographics, the types of CM agents used, experiences in managing contrast‐induced hypersensitivity reactions, and self‐reported proficiency in recognising and managing such reactions.

**Results:**

Ninety radiographers participated in the study. Most [80 (69.6%)] reported using non‐ionic low osmolar CM. The majority [82 (91.1%)] had received CM hypersensitivity reactions education as part of undergraduate training. Additionally, 63 (70.0%) radiographers received additional training. Most participants strongly agreed [67 (74.4%)] that radiographers play important roles in educating patients about CM and in reporting and documenting hypersensitivity reactions. Participants were of the view that history of prior hypersensitivity reactions to CM is the commonest patient factor to be associated with a higher risk of hypersensitivity contrast reactions during CT examinations [78 (48.4%)]. Forty‐three (47.8%) of the responses were of the view that screening patients for pre‐existing allergies as well as adequate prophylaxis and the use of lower‐risk contrast agents could minimise the occurrence of hypersensitivity contrast reactions. Only 34 (37.8%) and 18 (20.0%) of the participants indicated that they were either confident or highly confident in their ability to manage hypersensitivity reactions to CM respectively.

**Conclusion:**

The study revealed that radiographers may have a working knowledge of recognising and managing hypersensitivity reactions to CM. However, some radiographers were least confident in their ability to manage such reactions. This suggests the need for continuous training to ensure that radiographers are equipped with the necessary knowledge and skills for recognition and appropriate management of CM reactions.

## 1 Introduction

It is estimated that over 200,000 computed tomography (CT) examinations are performed yearly in Ghana, and a considerable amount involves the use of contrast media (CM) [1]. The increased utilisation of CT for diagnoses in Ghana [2, 3, 4] suggests an increase in the use of intravenous contrast agents for these examinations. With a total of more than 200,000 CT scans performed annually in Ghana and studies showing incidence of hypersensitivity reactions to CM being between 0.16% to 2.1% [5, 6, 7, 8], approximately 4,000 patients are expected to experience hypersensitivity reactions. Despite CM being considered safe, there is always a risk of adverse reactions. Hypersensitivity is a broad term encompassing both allergic‐like and physiologic contrast reactions. Allergic‐like reactions occur independently of the dose administered and can be caused by immunologic mechanisms. On the other hand, physiologic reactions are non‐allergic and dose‐dependent, often related to the chemical properties of the CM [26]. These reactions are characterised by nausea, vomiting, urticaria, pallor, skin oedema, erythema, wheezing, dyspnoea, rigour and laryngeal oedema [9, 10]. Additionally, signs such as hypotensive shock, pulmonary oedema, respiratory or cardiac arrest and convulsions may indicate severe CM reactions [11]. In CT imaging, health professionals assess the risk and potential benefit of contrast‐enhanced examination, and if justified, they screen patients carefully for any allergies and/or other medical conditions that may increase their risk of reacting to CM [12]. Patients are also informed about contrast agents' potential risks and benefits before they undergo the examination [12]. Therefore, preparation, such as properly trained individuals, equipment, medications, and pre‐arranged response planning, is necessary and should always be in place to mitigate possible CM reactions [13].

In Ghana, radiographers usually prepare and administer CM during CT examinations, including ensuring that adequate prophylaxis is provided to patients with higher risks of contrast‐induced hypersensitivity reactions. Again, the inadequate number of radiologists nationwide has resulted in radiographers having taken on the full responsibility of performing radiological investigations, including contrast‐enhanced CT procedures on their own. This means that in the absence of on‐site radiologists, radiographers are responsible for managing any hypersensitivity contrast reactions that may arise. However, radiographers in Ghana may have limited hands‐on experience in managing hypersensitivity reactions to CM, as such adverse events are rare and formal training in emergency response to contrast reactions is not consistently integrated into radiography curricula or clinical practice. Despite this, very few studies on the subject matter have focused on radiographers. Furthermore, there is a dearth of published studies on hypersensitive reactions associated with contrast CT in Ghana. This study, therefore, aims to investigate the experiences and self‐reported abilities of diagnostic radiographers in responding to contrast‐induced reactions associated with CT examinations.

## 2 Methods

### 2.1 Study Design

This study employed a prospective quantitative cross‐sectional electronic survey to investigate the experiences and self‐reported abilities of radiographers in managing acute contrast‐induced hypersensitivity reactions during CT examinations in Ghana. The study was reported in accordance with Strengthening the Reporting of Observational Studies in Epidemiology (STROBE) guidelines [14].

### 2.2 Study Setting

The Ghanaian population is estimated to be approximately 30.8 million people [15]. There are 16 regions, which include Volta, Oti, Greater Accra, Central, Western, Western North, Upper East, Upper West, Savannah, Ashanti, Eastern, Northern, North‐east, Brong Ahafo, Bono East, and Ahafo regions [16]. Radiographers play diverse and critical roles in the diagnosis of patients within the Ghanaian healthcare system. One of these diverse roles is performing CT examinations.

### 2.3 Study Respondents, Inclusion and Exclusion Criteria

The target population comprised radiographers in active clinical service. To be eligible for the study, radiographers ought to have been licensed by the Allied Health Professions Council of Ghana (AHPC) and routinely perform CT examinations. Radiographers holding provisional licenses, intern radiographers on mandatory clinical rotations, radiographers not in active clinical practice, and those not performing CT examinations were excluded from the study.

### 2.4 Sample Size

A study by Botwe et al. [1] revealed 107 qualified practising CT radiographers in Ghana. Using the Taro Yamane 1967 [17] sample size formula for computation, a minimum sample size of 85 was calculated. Subsequently, a non‐probability purposive sampling method was used to recruit radiographers for the study. This method was chosen due to the small nature of the study population: it allowed the researchers to access CT radiographers quickly and efficiently.

### 2.5 Questionnaire and Data Collection

A questionnaire was used to collect data from radiographers who met the eligibility criteria. The questionnaire was developed by the authors after reviewing relevant literature on the topic [5, 9, 13, 18]. To ensure the reliability and appropriateness of the questionnaire, three experienced radiography academics were invited to review the draft questionnaire. Subsequently, five radiographers with at least 2 years of experience in routinely performing CT examinations were invited to fill out the questionnaire as a pilot study. Feedback from the pilot study was used to modify the questionnaire before the main data collection commenced. Results from the pilot study were excluded from the main study. The data collection instrument was structured into four sections: first section covered information on participant's demographics, second section on common CM agents used by radiographers as well as their education in managing hypersensitivity contrast reactions. The third section covered questions about radiographers' experiences in the management of contrast hypersensitivity reactions associated with CT examinations. The final section of the questionnaire evaluated the perception of participants on the role radiographers can play in preventing, recognising and managing hypersensitivity contrast reactions. An open‐ended question was also included, allowing radiographers to respond to the options that may not have been included in the alternatives provided. All questions, except for the follow‐up and open‐ended items, were mandatory. The link to the Google form questionnaire was distributed via the email addresses of the participants by the Ghana Society of Radiographers (GSR) on behalf of the researchers. The data was collected over a period of 6 months. The questionnaire was designed to allow each respondent to complete the questionnaire once by first logging in with their email addresses. After they had completed and submitted the questionnaire, they were not allowed to log in using the same email address. This helped to avoid duplication of results.

### 2.6 Data Analysis

The quantitative data from the questionnaires was fed into the IBM SPSS version 26 (IBM Corp., Armonk, NY, USA). The results were analysed using descriptive statistics in the form of charts, frequency tables, and percentages. Chi‐square analysis was used to test for differences between receiving undergraduate education on CM hypersensitivity reactions and radiographers' ability to recognise and manage such reactions. It was also used to examine the relationship between obtaining additional training or education on handling CM allergic reactions and radiographers' ability to recognise and manage hypersensitivity reactions to CT CM. A *p*‐value of less than 0.05 significant level was considered statistically significant at a 95% confidence interval.

### 2.7 Ethics Approval and Consent to Participate

The study received ethical approval from the Research and Ethics Committee of the University of Health and Allied Sciences (UHAS‐REC A.8 [94] 22‐23), as well as from the GSR (GSR/NEC/GS/RS‐01/07/23). An information sheet describing the purpose of the study was attached to the questionnaire. Participation was voluntary and all participants provided electronic informed consent for participation. All the data obtained from participants were kept anonymous, encrypted, and then stored on a password‐protected laptop which was only accessible by the authors.

## 3 Results

### 3.1 Demographic Characteristics

Out of the 107 questionnaires distributed, a total of 90 radiographers responded and filled out the questionnaire. The majority of the participants were males [67 (74.4%)], and most held a bachelor's degree as their highest level of education [59 (65.6%)]. The ages of the participants ranged from 20 to 56 years with majority [41 (45.6%)] within the 20–30‐year age group. Additionally, 56 (62.2%) participants were employed in public healthcare facilities with the majority [40 (44.4%)] located within the Greater Accra Region. Table 1 provides details of the radiographers' demographic characteristics.

**Table 1:** Radiographers' demographic distribution.

Variable	Category	Frequency (*N*)	Percentage %
Gender	Male	67	74.4
	Female	23	25.6
Age (years)	20–30	41	45.6
	31–40	24	26.7
	Above 40 years	25	27.8
Level of education	Diploma	5	5.6
	Bachelor's degree	59	65.6
	Master's degree	24	26.7
	PhD	2	2.2
Years of work experience	Below 5 years	33	36.7
	5–10 years	17	18.9
	Above 10 years	40	44.4
Type of facility	Public	56	62.2
	Private	34	37.8
Region of facility	Greater‐Accra	40	44.4
	Ashanti	19	21.1
	Northern	6	6.7
	Central	6	6.7
	Western	5	5.6
	Bono East	3	3.3
	Eastern	3	3.3
	Upper West	3	3.3
	Volta	3	3.3
	Savannah	2	2.2
	Upper East	0	0.0
	Bono	0	0.0
	Ahafo	0	0.0
	North‐East	0	0.0
	Western North	0	0.0
	Oti	0	0.0

### 3.2 Common Contrast Agents Used by Radiographers

Most participant radiographers [80 (88.9%)] reported frequent use of iodinated CM, specifically non‐ionic low‐osmolar agents, for contrast‐enhanced CT examinations. Iohexol was identified as the most commonly administered intravenous contrast medium [71 (78.9%)], while Iodixanol was the least utilized [1 (1.1%)]. Similarly, Iohexol was the most frequently used oral contrast agent [59 (65.6%)], Table 2 presents the detailed distribution of contrast agents used in CT studies as reported by the respondents.

**Table 2:** Common contrast agents used for CT studies.

Variable	Category	Frequency	Percentage
Type of CM used by radiographers	Iodinated CM (Non‐ionic low osmolar)	80	88.9
	Iodinated CM (Non‐ionic iso‐osmolar)	21	23.3
	Iodinated CM (Ionic high osmolar)	14	15.6
Intravenous CM used by radiographers	Iohexol	71	78.9
	Iopamidol	45	50
	Iodipamide meglumine	2	2.2
	Sodium diatrizoate	2	2.2
	Iodixanol	1	1.1
Oral CM used by radiographers	Iohexol	59	65.6
	Sodium amidotrizoate and meglumine amidotrizoate	24	26.7
	Iopamidol	15	16.7
	Mannitol	2	2.2

### 3.3 Radiographers' Education in Managing Hypersensitivity Contrast Reactions

**Figure 1:**
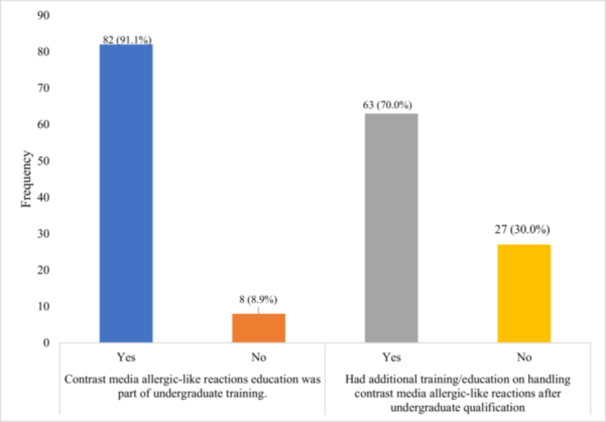
Radiographers' education with hypersensitivity contrast reactions.

The vast majority of radiographers [82 (91.1%)] reported receiving education on CM hypersensitivity reactions during their undergraduate training. Additionally, 63 radiographers (70.0%) had received further training on hypersensitivity contrast reactions beyond their undergraduate education, as shown in Figure 1.

**Figure 2:**
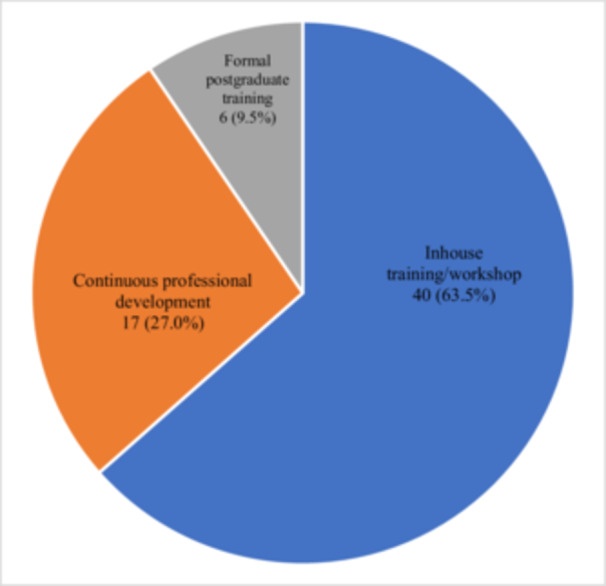
Type of additional training received on hypersensitivity contrast reactions.

Among those who had received additional training, 40 (63.5%) reported participating in in‐house training or workshops. A detailed breakdown of the types of additional training is presented in Figure 2.

### 3.4 Radiographer's Experiences With Hypersensitivity Contrast Reactions

Concerning the frequency of hypersensitivity reactions, 46 (51.1%) participants indicated that hypersensitivity contrast reactions rarely occur. Forty‐two (46.7%) participants also reported that hypersensitivity contrast reactions occur immediately or within a few minutes. Table 3 shows radiographers' experiences with hypersensitivity contrast reactions.

**Table 3:** Radiographers' experiences with hypersensitivity contrast reactions.

Variable	Category	Frequency	Percentage
Encountered patients exhibiting hypersensitivity contrast reactions during contrast CT examinations?	Yes	63	70.0
	No	27	30.0
Frequency of observed hypersensitivity contrast reactions in patients during contrast CT examinations	Rarely	46	51.1
	Occasionally	17	18.9
	Frequently	0	0.0
Time of occurrence of hypersensitivity contrast reaction observed by the radiographers.	Immediately	42	46.7
	Within 30 min	15	16.7
	Within an hour	5	5.6
	After an hour	0	0.0
	Days/weeks after	1	1.1
Observed any trends or patterns in the occurrence of hypersensitivity contrast reactions in certain patient populations or demographics	Yes	11	12.2
	No	52	57.8
Patient populations or demographics observed to be more prone to hypersensitivity contrast reactions during contrast CT examinations	Paediatrics patients	1	1.1
	Geriatric patients	1	1.1
	Patients with pre‐existing allergies	8	8.9
	Patients with renal impairment	1	1.1
Patients are informed to report hypersensitivity contrast effects even after days or weeks after the CT contrast examination	Strongly agree	38	42.2
	Agree	27	30.0
	Neutral	13	14.4
	Disagree	11	12.2
	Strongly disagree	1	1.1
Frequency of patients reporting late hypersensitivity contrast reactions	Never	22	24.4
	Rarely	58	64.4
	Occasionally	10	11.1
	Frequently	0	0.0
Mild hypersensitivity contrast reactions	Nausea or vomiting	59	65.6
	Metallic taste in the mouth	33	36.7
	Itching or hives	19	21.1
	Flushing or feeling warm	42	46.7
	Dizziness	24	26.7
Moderate hypersensitivity contrast reactions	Swollen face, lips and tongue	7	7.8
	Shortness of breath	20	22.2
	Moderate itching and tingling in the mouth or throat	24	26.7
	Elevated heart rate or blood pressure	7	7.8
Severe hypersensitivity contrast reactions	Seizures	7	7.8
	Hypotension	22	24.4
	Cardiac arrest	5	5.6
	Bronchospasm	7	7.8
	Angioedema	4	4.4

### 3.5 Perception of the Role Radiographers Play in Preventing, Recognising and Managing Hypersensitivity Contrast Reactions

Most participants strongly agreed [67 (74.4%)] that radiographers play important roles in educating patients about CM and in reporting and documenting hypersensitivity reactions during CT examinations. According to the participants, they perceived ‘history of prior hypersensitivity reactions to CM’ as the commonest patient factor to be associated with a higher risk of hypersensitivity contrast reactions during CT examinations [78 (86.7%)]. Forty‐three (47.8%) of the participants were of the view that screening patients for pre‐existing allergies could minimise the occurrence of hypersensitivity contrast reactions, as well as adequate prophylaxis and the use of lower‐risk contrast agents. Additionally, almost all participants [87 (94.6%)] believed that improved education and training for radiographers could further improve the recognition and management of hypersensitivity (hypersensitivity) contrast reactions in patients undergoing CT examinations. Table 4 shows the perception of the role radiographers can play in preventing, recognising and managing hypersensitivity contrast reactions.

**Table 4:** Perception of the role radiographers can play in preventing, recognising, and managing hypersensitivity contrast reactions.

Variable	Category	Frequency	Percentage
Radiographers play an important role in educating patients about CM and in reporting and documenting hypersensitivity reactions during CT examinations	Strongly agree	67	74.4
	Agree	21	23.3
	Neutral	2	2.2
	Disagree	0	0.0
	Strongly disagree	0	0.0
Generally, our unit routinely evaluates patients for risk factors and their allergy history before administering CM for a CT examination	Strongly agree	58	64.4
	Agree	20	22.2
	Neutral	9	10.0
	Disagree	1	1.1
	Strongly disagree	2	2.2
In your experience, which of the following patient factors have been associated with a higher risk of hypersensitivity contrast reactions during CT examinations?	History of prior hypersensitivity reactions to CM	78	86.7
	History of asthma or other respiratory conditions	56	62.2
	Age	21	23.3
	Gender	1	1.1
	Diabetic patients taking metformin	1	1.1
	History of seafood allergy	1	1.1
	Low GFR (Glomerulus Filtration Rate)	1	1.1
	Eating shortly before the procedure	1	1.1
	Fasting a few hours to the procedure	1	1.1
In your experience, what are the most effective preventive measures that can be taken to minimize the occurrence of hypersensitivity (hypersensitivity) contrast reactions during contrast CT examinations?	Pre‐medication with antihistamines	20	22.2
	Use of lower‐risk contrast agents	26	28.9
	Screening patients for pre‐existing allergies	43	47.8
	Communication	1	1.1
How do you typically manage patients who exhibit hypersensitivity (hypersensitivity) contrast reactions during CT examinations?	Discontinue the contrast injection	64	71.1
	Administer antihistamines or corticosteroids	52	57.8
	Substitute ionic with non‐ionic contrast	13	14.4
	Provide oxygen therapy or assist with breathing	27	30.0
	Contact the radiologist or physician for further instructions	0	0.0
	Never managed hypersensitivity contrast reactions	1	1.1
What steps do you believe can be taken to further improve the recognition and management of hypersensitivity (hypersensitivity) contrast reactions in patients undergoing CT examinations?	Improved education and training for radiographers	87	94.6
	Improved communication between radiographers and other healthcare providers	63	70.0
	Standardised protocols for managing hypersensitivity reactions	79	87.8
	Improved patient education and informed consent processes	78	86.7

### 3.6 Self‐Reported Abilities of Diagnostic Radiographers in Responding to Contrast‐Induced Allergic Reactions

The radiographers were asked to rate their confidence in their ability to recognise and manage hypersensitivity reactions to CT CM and how well their department and/or colleagues were prepared to respond to and manage hypersensitivity contrast reactions in patients during contrast CT examinations. Fifty‐two (57.8%) radiographers indicated they were confident or highly confident in their ability to recognise and manage hypersensitivity reactions to CT CM. A significant association was observed between receiving CM hypersensitivity reactions as part of undergraduate education and radiographers' ability to recognise and manage hypersensitivity reactions to CT CM [*χ*²(1, *N* = 90) = 22.24, *p* > 0.05]. However, there was no significant association between obtaining additional training/education on the handling of CM hypersensitivity reactions and radiographers' ability to recognise and manage hypersensitivity reactions to CT CM [*χ*²(1, *N* = 90) = 6.29, *p* < 0.05]. Table 5 shows a comparison between radiographers' level of confidence in recognising and managing hypersensitivity reactions to CT CM and training.

**Table 5:** Association between radiographers' level of confidence in recognising and managing hypersensitivity reactions to CT CM and training.

Variable 1 – CM hypersensitivity reactions education was part of undergraduate training.					
Variable	Rating	Yes *n* (%)	No *n* (%)	Total *n* (%)	*p* value
Ability to recognise and manage hypersensitivity reactions to CT CM	1 – Least confident	1 (1.2)	1 (12.5)	2 (2.2)	**0.03**
	2	7 (8.8)	2 (25.0)	9 (10.0)	
	3	23 (28.0)	4 (50.0)	27 (30.0)	
	4	33 (40.2)	1 (12.5)	34 (37.8)	
	5 – Highly confident	18 (20.0)	0 (0.0)	18 (20.0)	
	**Total**	**82 (91.1)**	**8 (8.9)**	**90 (100.0)**	
**Variable 2 – Had additional training/education on handling CM hypersensitivity reactions after undergraduate qualification.**					
Ability to recognise and manage hypersensitivity reactions to CT CM	1 – Least confident	1 (1.6)	1 (3.7)	2 (2.2)	**0.13**
	2	4 (6.3)	5 (18.5)	9 (10.0)	
	3	17 (27.0)	10 (37.0)	27 (30.0)	
	4	25 (39.7)	9 (33.3)	34 (37.8)	
	5 – Highly confident	16 (25.4)	2 (7.4)	18 (20.0)	
	**Total**	**63 (70.0)**	**27 (30.0)**	**90 (100.0)**	

## 4 Discussion

The study investigated the experiences and self‐reported abilities of diagnostic radiographers in responding to contrast‐induced hypersensitivity reactions associated with CT examinations. CM Per the experiences of the radiographers of this study, most of the hypersensitivity reactions are mild with nausea or vomiting as the most common mild hypersensitivity reaction [59 (65.6%)]. For moderate hypersensitivity reactions, moderate itching or tingling in the mouth or throat was the most common [24 (26.7%)] while hypotension was the most frequent severe hypersensitivity contrast reaction [22 (24.4%)] (Table 3). The findings agree with a study in Japan by Kobayashi et al. [27] which showed that, nausea and/or vomiting was the most frequent mild hypersensitivity contrast reaction (31.8%). Contrarily, urticaria was the most common mild hypersensitivity reaction (51.70%) in a study done in China by Li et al. [28] with nausea or vomiting being the second most common mild hypersensitivity reaction. In that same study, hypotension was the commonest severe hypersensitivity contrast reaction (19.57%) which agrees with this study. The finding that most hypersensitivity reactions to CM are mild suggests that radiographers should be well‐prepared to manage these minor reactions promptly to prevent escalation. However, the occurrence of severe reactions such as hypotension highlights the importance of maintaining emergency preparedness and ensuring immediate access to resuscitation equipment and trained personnel during contrast‐enhanced procedures.

The findings revealed that non‐ionic, low osmolar iodinated CM were the most common contrast agents administered intravenously during CT procedures (Table 2). This aligns with a study conducted by Jimah et al. [2] which reported Iohexol – a non‐ionic low osmolar iodinated CM as the sole contrast agent used for head CT examinations in Ghana. The preference for non‐ionic low osmolar iodinated CM, as indicated in this study may be attributed to their numerous advantages. These contrast agents are associated with a significantly lower risk of adverse reactions, such as hypersensitivity and nephrotoxicity, compared to their high‐osmolar counterparts [16]. Additionally, their use enhances patient comfort by minimising the sensation of warmth and discomfort during administration. Furthermore, their versatility makes them suitable for a wide range of patient populations, including those with pre‐existing conditions such as renal impairment, allergies, or cardiovascular diseases. This broad applicability may explain why they are often the contrast agents of choice for CT examinations [17, 18]

The study also indicated that training on handling CM reactions was largely received by the respondents during their undergraduate studies and from in‐house training/workshops. Even though this may be enough to handle mild hypersensitivity contrast reactions, further review of responses from respondents suggests the need for further in‐depth education/training. For instance, just a few [11 (12.2%)] (Table 3) radiographers ever mention having observed any trends in the occurrence of hypersensitivity contrast reactions in certain patient populations even though there is a wealth of knowledge on the subject matter. According to Wang et al. [19] and Trygg et al. [9] confidence in handling hypersensitivity reactions improves as radiographers encounter and manage more severe cases. This underscores the importance of regular simulation‐based training, ideally at intervals of 6 to 12 months [20] on handling severe reactions to boost confidence and better prepare radiographers for real‐life scenarios to enhance radiographers' preparedness and confidence in responding to severe reactions in real‐life clinical scenarios. Studies show that cardiopulmonary resuscitation (CPR) is an important management strategy that helps in the advent of severe contrast reactions such as cardiac arrest [29,30] hence it is important that radiographers practice CPR Additionally, most of the radiographers reported that most hypersensitivity reactions typically occur immediately or within a few minutes after the administration of CM (Table 3). This is consistent with previous studies [21, 22]. This may be because delayed reactions often occur outside the radiographers' direct observation, and patients may not report these hypersensitivity reactions, possibly due to a lack of information or awareness that such delayed responses can occur [22].

In managing patients who exhibit hypersensitivity contrast reactions during CT examinations, radiographers reported that they mostly discontinued the contrast examination and/or administered antihistamines or corticosteroids. These findings are in agreement with the propositions of the American College of Radiology [13]. The ACR recommends the documentation of information such as the precise name and dose of the contrast medium, the manifestations (symptoms and signs) of the reaction and the chronology of the reaction and the specific treatment given [23]. It is worth noting that none of the radiographers mentioned managing contrast reactions by contacting a radiologist or physician for further instructions. This may be due to the few radiologists in Ghana resulting in radiographers having to take on the full responsibility of performing radiological investigations, including contrast‐enhanced CT procedures on their own [24]. This highlights the need for radiographers to have the knowledge and skills to administer the needed medication or adequate prophylaxis to treat or prevent hypersensitivity reactions during contrast CT examinations. Consistent with previous studies [9, 25], most radiographers feel they need improved education including improved communication between radiographers and other healthcare providers, standardised protocols for managing hypersensitivity reactions, and improved patient education and informed consent processes. Comparing radiographers' level of confidence in recognising and managing hypersensitivity reactions to CT CM and training received, the study showed that most radiographers who reported having received training during their undergraduate education or had additional training had higher self‐reported confidence levels (Table 5). Even though more than half of the participants rated themselves to be confident or highly confident in their ability to manage CM reactions, about 40% of participants rated themselves lower. This finding emphasises the significance of training and re‐training, as these activities enhance confidence levels [9].

### 4.1 Limitations of the Study

The data collected for this study relies on self‐reported information from diagnostic radiographers hence, the potential for recall bias or social desirability bias cannot be ruled out, as participants may not accurately report their experiences or may provide answers they believe are expected. This suggests a need for further study such as an observational or qualitative study focused on objectively exploring the practices of diagnostic radiographers in managing these reactions.

## 5 Conclusion

This study successfully addressed its aim of investigating the experiences and self‐reported abilities of diagnostic radiographers in responding to contrast‐induced reactions associated with CT examinations. The findings indicate that while radiographers encounter various categories of hypersensitivity contrast reactions, their ability to recognize and manage these reactions is hindered by gaps in training and competence, particularly for severe reactions. To improve patient outcomes, regular and modernized training, such as simulation‐based learning, is essential to enhance radiographers' skills and confidence. Furthermore, a multidisciplinary approach involving radiologists is recommended, suggesting that a similar study be conducted among radiologists to foster a cohesive and effective response to contrast hypersensitivity reactions.

## Author Contributions

**Vivian Della Atuwo‐Ampoh:** conceptualization, investigation, methodology, supervision, writing – review and editing, writing – original draft, validation, resources, project administration. **Paul Kasem:** investigation, methodology, writing – review and editing, writing – original draft, validation, data collection, formal analysis. **Patience Addo:** investigation, methodology, supervision, writing – review and editing. **Bismark Ofori‐Manteaw:** writing – review and editing, formal analysis, supervision, data curation. **Justice Hanson:** methodology, data collection, resources, writing – review and editing. **David Adedia:** formal analysis, software, data curation, validation, methodology, writing – review and editing. **Nathaniel Awentiirin Angaag:** investigation, writing – original draft, writing – review and editing, data curation, methodology. **Jacob Leonard Ago:** writing – review and editing, formal analysis, validation, visualization. **Klenam Dzefi‐Tettey:** supervision, visualization, project administration, conceptualization.

## Funding

The authors received no specific funding for this work.

## Conflicts of Interest

The authors declare no conflicts of interest.

## 1 Transparency Statement

The lead author (Vivian Della Atuwo‐Ampoh) affirms that this manuscript is an honest, accurate, and transparent account of the study being reported; that no important aspects of the study have been omitted; and that any discrepancies from the study as planned (and, if relevant, registered) have been explained.

## Data Availability

The data supporting the findings of this study are available from the corresponding author upon reasonable request. The data are not publicly available due to privacy or ethical restrictions.
